# Simple Renal Cysts in Marfan Syndrome: Prevalence and Association With Aortic Events

**DOI:** 10.1016/j.jacadv.2025.101870

**Published:** 2025-06-17

**Authors:** Claire Bouleti, Noemie Tence, Raphael Thuillier, Florence Nicot, Nicoletta Pasi, Benjamin Alos, Gaspard Suc, Olivier Milleron, Florence Arnoult, Maria Tchitchinadze, Maud Langeois, Catherine Boileau, Laurent Gouya, Phalla Ou, Guillaume Jondeau

**Affiliations:** a University of Poitiers, Clinical Investigation Center (INSERM 1402), FACT and Poitiers Hospital, Cardiology Department, Poitiers, France; b Private Hospital Jacques Cartier, Cardiology Department, Massy, France; c Inserm Unit Ischémie Reperfusion, Métabolisme et Inflammation Stérile en Transplantation (IRMETIST), UMR U1313, Poitiers, France; d Faculty of Medicine and Pharmacy, University of Poitiers, Poitiers, France; e Biochemistry Department, CHU Poitiers, Poitiers, France; f Hospital of Versailles, Cardiology Department, Le Chesnay, France; g Private Hospital Paul Degine, Radiology Department, Champigny-sur-Marne, France; h Bichat University Hospital, AP-HP, Cardiology Department, Paris, France; i Bichat University Hospital, AP-HP, Reference Center for Marfan Disease, Cardiology Department, U1148 LVTS, INSERM, Université Paris Cité, Paris, France; j University Hospital of Toulouse, Genetic Department, Toulouse, France; k Bichat University Hospital, AP-HP, Reference Center for Marfan Disease, Genetic Department, Université Paris Cité, Paris, France; l Bichat University Hospital, AP-HP, Genetic Department, U1148 LVTS, INSERM, Université Paris Cité, Paris, France; m Bichat University Hospital, AP-HP, Radiology Department, Paris, France

**Keywords:** CT scan, Marfan syndrome

## Abstract

**Background:**

A link between simple renal cysts (SRCs) and aortic aneurysms or dissection has been reported in the general population. Marfan syndrome (MFS) is associated with severe aortic disease, but very few data on SRCs exist in this population.

**Objectives:**

The objectives were to evaluate: 1) the prevalence of SRCs in patients with MFS, compared to matched controls; and 2) the association between SRCs and aortic events in patients with MFS.

**Methods:**

Consecutive patients with MFS ascertained by a pathogenic variant in the fibrillin-1 gene who underwent complete computed tomography scans at our institution were included and matched 1:1 for age and sex with controls.

**Results:**

Between 2010 and 2016, 131 patients with MFS and 131 controls were included. The mean age was 40 ± 14 years, with 42% women. SRC prevalence was higher in patients with MFS: 41% vs 21% in controls (*P* < 0.0001). SRC prevalence increased with aortic disease severity: 59% in patients with dissection, 43% in patients with aortic aneurysm surgery, and 19% in patients with MFS but without aortic events, similar to 21% in controls (*P* < 0.009). In multivariable analysis, SRC presence in patients with MFS was independently associated with aortic dissection (adjusted OR: 2.30 [95% CI: 1.00-5.32]; *P* = 0.049).

**Conclusions:**

The prevalence of SRCs was significantly higher in patients with MFS compared to matched controls. SRCs were independently associated with aortic dissection in MFS. Prospective studies are needed to further evaluate whether SRCs could represent a marker of aortic disease severity in patients with MFS.

Simple renal cysts (SRCs) are common in the general population and their prevalence increases with age, with 50% of patients aged ≥60 years having at least 1 renal cyst in computed tomography (CT) scan screening series.^1^ A link between SRCs and aortic diseases has been reported by different series, both for aortic aneurysm and dissection.^2^, ^3^, ^4^ Several hypotheses have been proposed, including an increased activity of matrix metalloproteinases (MMPs) leading to a structural vulnerability^2^^,^^5^^,^^6^ or enhanced inflammatory activity,^7^ but no clear common pathophysiology is elucidated so far.

Marfan syndrome (MFS) is due to a pathogenic variant in the fibrillin-1 (*FBN1*) gene that encodes for a key protein of the extracellular matrix (ECM).^8^^,^^9^ *FBN1* variants lead to a weakening of the connective tissues and are associated with an increased activity of MMPs and the dysregulation of transforming growth factor-beta (TGF-β).^10^^,^^11^ The main source of mortality in MFS is the dilation of the aorta, with an increased risk of aortic and extra-aortic dissection.^12^, ^13^, ^14^, ^15^

Although patients with MFS often present with severe aortic diseases, no study has focused on the association between MFS due to *FBN1* pathogenic variants and SRCs.

The aims of this paper were to: 1) evaluate the prevalence of SRCs in patients with MFS confirmed by proven *FBN1* pathogenic variants, compared to matched controls without MFS; and 2) assess the prevalence of SRCs in relation to the severity of aortic disease (aneurysm requiring surgery or dissection) in MFS.

## Methods

### Population

**Figure 1:**
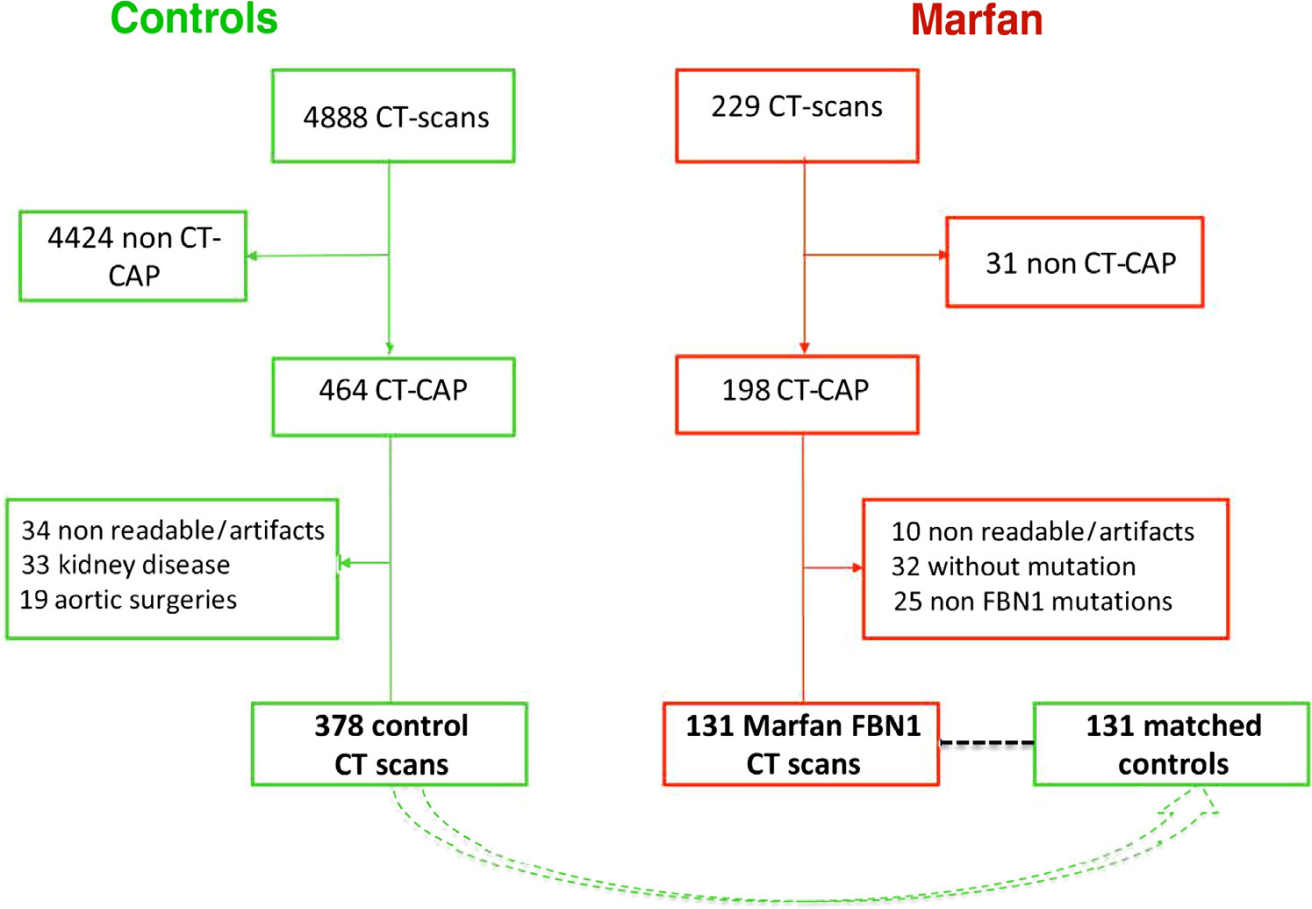
Flow Chart of the Study Of the 229 patients with a diagnosis of MFS who underwent a contrast-enhanced CT scan at our institution, 31 had a CT scan that did not include the entire kidneys and were thus excluded from the study. Among the 198 patients with a CT-CAP, 67 were excluded due to poor quality of images with non-ECG gating (n = 8), having only 1 kidney (n = 2), no identified pathogenic variant (n = 32), pathogenic variants in genes other than *FBN1* (n = 25). Ultimately, 131 patients with MFS were included in the study. Of 4,888 control patients who underwent a CT scan, 464 had a CT-CAP. Among them, 96 patients were excluded due to poor quality of the images in 34 (no ECG gating or severe artifacts), kidney diseases in 33, and previous aortic surgery in 19. Finally, among these 378 control patients, 131 were matched with patients with MFS. CT = computed tomography; CT-CAP = CT of the chest, abdomen, and pelvis; ECG = electrocardiogram; FBN1 = fibrillin-1; MFS = Marfan syndrome.

We retrospectively included consecutive patients treated at our institution (the National Reference Center for Marfan disease and related disorders at Bichat Hospital) according to the following criteria (Figure 1): adult patients diagnosed with MFS with a confirmed pathogenic variant in the FBN1 gene. Exclusion criteria were patients under 18 years of age and those without a contrast-enhanced CT scan allowing proper analysis of the kidneys.

Age- and sex-matched controls were selected from the database of CT scans conducted at the same center (Bichat Hospital) among adult patients who underwent contrast-enhanced CT scans of the chest, abdomen, and pelvis (CT-CAP) enabling kidney analysis, and without any known aortic disease. Clinical data for patients with MFS were collected during multidisciplinary visits and data for control patients were obtained from their medical records.

This study was conducted in accordance with the principles of the Declaration of Helsinki and meets the STARD requirements. The study obtained Human Study IRB approvals (n°1229912 and n°2207326) and was registered in the Institut National des Données de Santé (MR3310200918) as required by local regulations.

Regarding the prevalence of SRCs in patients with MFS and controls, we initially aimed for a minimum of 105 patients in each group to achieve 90% power of analysis with an alpha of 0.05 (power calculations conducted using the pROC package), based on the published OR of 2.78 in the general population.^3^ Therefore, the final population of 131 patients in each group ensures appropriate power for our analysis.

### CT scan: kidneys and renal cysts analysis

Only high-quality contrast-enhanced, electrocardiogram-gated CT-CAP scans were selected, allowing for the analysis of both kidneys. CT scans were performed at our institution using a 64-slice machine (GE Discovery CT 750HD).

A renal cyst was defined as a thin-walled, circular lesion filled with fluid, without evidence of septations, and measuring at least 2 mm, as previously reported^16^ (Supplemental Figure 1). The number of cysts in each kidney as well as the length and width of both kidneys were analyzed for each patient.

All measurements were performed by 2 senior radiologists.

### Aortic aneurysm

Aortic measures were made using 2 dimensions imaging at end diastole, in a strictly perpendicular plane to that of the long axis of the aorta using the leading-edge to leading-edge convention for aortic root and the ascending aorta.^17^^,^^18^ Aortic diameters (root and ascending aorta) were converted to *z*-scores, correcting for age, body surface area, and sex, using the Campens method.^19^ Aortic aneurysm was defined as a *z*-score ≥2 indicating a value exceeding 2 SDs (the 95% CI) as recommended.

### Statistical analysis

Quantitative variables were expressed as mean ± SD or median (25th-75th percentiles) as appropriate, while qualitative variables were expressed as numbers and percentages. Comparisons between matched groups were performed using paired *t*-tests or Wilcoxon rank tests for quantitative variables, as appropriate, and Mc Nemar chi-square tests for qualitative variables. Comparisons between independent groups were performed using *t*-tests or Mann-Whitney *U* tests for quantitative variables, as appropriate, and chi-square tests for qualitative variables. Concordance between the 2 blinded physicians regarding SRC presence was assessed using a Kappa test. Logistic regression was used to analyze the risk of dissection in patients with MFS. Variables with *P* value <0.15 in univariate analysis were further included in the multivariable analysis. To investigate the interaction between dissection and the presence of SRC, we used the finalfit package in R to build a generalized linear model explaining the occurrence of dissection. This model incorporated the presence of SRC along with other parameters known to be associated with dissection (listed in Supplemental Table 1). To assess collinearity, we used the variance inflation factor (Supplemental Table 1) and verified correlations using the Spearman correlation test (Supplemental Tables 2 and 3).

Results were considered significant when 2-sided *P* values were <0.05.

Analyses were performed using SPSS statistical software (SPSS V.23, SPSS Inc) and R software (R Core Team. R: A Language and Environment for Statistical Computing. 2023).

## Results

### Population

Between June 2010 and January 2016, a total of 229 patients with MFS who underwent a contrast-enhanced CT scan at our institution were identified. After excluding patients per protocol, 131 consecutive patients with a pathogenic variant in the *FBN1* gene and reliable CT-CAP imaging were included, defining the MFS study population. The indications for CT-CAP were as follows: follow-up after aortic surgery and/or dissection (n = 46), aortic diameters close to the surgical threshold and/or rapid progression between 2 echocardiographic examinations (n = 58), first CT scan for reference imaging (n = 24), and chest pain with unconfirmed suspicion of aortic dissection or pneumothorax (n = 3).

Between October and December 2015, 4,888 control patients underwent CT scans in our institution, of whom 464 patients fulfilled the criteria for contrast-enhanced CT-CAP. After exclusion of patients per protocol, 378 controls were identified, and 131 were matched 1:1 for age and sex with patients with MFS.

The detailed population flow chart is presented in Figure 1.

The characteristics of patients with MFS and their matched controls are detailed in Table 1.

**Table 1:** Baseline Characteristics of the 131 MFS Patients and Their Matched Controls

	MFS (n = 131)	Controls (n = 131)	*P* Value
Age (y)	39.8 ± 13.8	39.7 ± 13.8	0.98
Female	55 (42)	55 (42)	1.00
Height (cm)	185 ± 11	171 ± 9	<0.0001
Weight (kg)	80.3 ± 16.6	71.3 ± 19.8	<0.0001
Body mass index (kg/m^2^)	23.5 ± 4.4	24.6 ± 6.6	0.18
Body surface area (m^2^)	2.02 ± 0.24	1.82 ± 0.24	<0.0001
Systolic BP (mm Hg)	128 ± 15	125 ± 22	0.20
Diastolic BP (mm Hg)	73 ± 10	76 ± 13	0.15
Heart rate (beats/min)	62 ± 13	85 ± 18	<0.0001
Smoking	5 (4)	38 (29)	<0.0001
Dyslipidemia	3 (2)	6 (5)	<0.0001
Diabetes	1 (1)	13 (10)	<0.0001
ARBs/ACEi	30 (23)	8 (6)	<0.0001
Beta-blockers	109 (83)	9 (7)	<0.0001

Values are mean ± SD or n (%).

ACEi = angiotensin-converting enzyme inhibitor; ARBs = angiotensin II receptor blockers; BP = blood pressure; MFS = Marfan syndrome.

Patients with MFS had a significantly higher body surface area, with taller height and heavier weight, as compared with controls. They had significantly less cardiovascular risk factors than their matched controls. They had a significantly lower heart rate due to the important proportion of patients treated with beta-blockers (83% vs 7%; *P* < 0.001), as recommended.^20^^,^^21^

### Prevalence of SRC in MFS and control groups

Among the 262 patients analyzed, both readers identified 178 patients without SRC and 73 patients with ≥1 SRC, with excellent interobserver agreement, as indicated by a Kappa value of 0.90.

CT scan analyses of renal parameters for both groups are detailed in Table 2.

**Table 2:** CT Scan Parameters Regarding Kidneys and SRC in MFS Patients and Controls

	MFS Patients (n = 131)	Controls (n = 131)	*P* Value
Right kidney width (mm)	52.2 ± 7.5	52.2 ± 6.3	0.99
Right kidney length (mm)	108.9 ± 11.8	111.6 ± 11.8	0.08
Left kidney width (mm)	54.5 ± 7.2	55.1 ± 6.8	0.44
Left kidney length (mm)	114.1 ± 18.4	112.9 ± 12.5	0.60
≥1 SRC	54 (41)	27 (21)	<0.0001
≥2 SRC	25 (19)	12 (9)	0.05
Bilateral SRC	15 (11)	10 (8)	0.4
Mean maximal diameter of SRC (mm)	16.3 ± 17.7	17.9 ± 15.8	0.38

Values are mean ± SD or n (%).

CT = computed tomography; SRC = simple renal cyst; other abbreviation as in Table 1.

The length and width of the kidneys were not significantly different between the 2 groups.

The presence of SRC was twice as frequent in patients with MFS compared to their matched controls: 54 patients with MFS with at least 1 cyst (42%) vs 27 controls (21%) (*P* < 0.0001). The proportion of patients with at least 2 SRCs was also higher in patients with MFS compared to controls, while the prevalence of bilateral cysts was not statistically different between the 2 groups. The mean maximal diameter of SRCs was not significantly different between groups.

Patients with SRCs were significantly older in both groups compared to those without cysts. In patients with MFS, the mean age was 44 ± 14 years in those with SRCs vs 37 ± 13 years in those without (*P* = 0.005). In controls, the mean age was 47 ± 16 years in those with SRCs vs 38 ± 13 in those without (*P* = 0.005).

**Figure 2:**
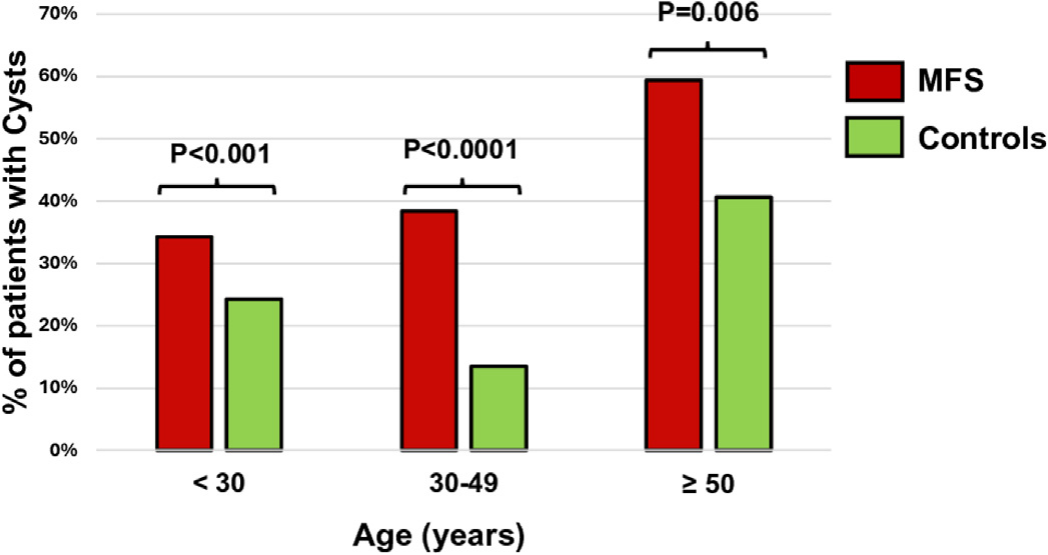
SRC Prevalence in Patients With MFS and Their Matched Controls According to Age Categories SRC = simple renal cyst; other abbreviation as in Figure 1.

Given the relationship between age and SRC prevalence, we analyzed the proportion of patients with SRCs in both groups according to age categories, as illustrated in Figure 2. The prevalence of SRCs increased with age but remained higher in patients with MFS compared to their matched controls. Indeed, in patients aged <30 years, the prevalence of SRCs was 33% in patients with MFS (12/36) vs 17% in controls (6/36) (*P* < 0.001). In patients aged 30 to 49 years, it was 38% in patients with MFS (23/61) vs 12% in controls (7/61) (*P* < 0.0001). Finally, in patients aged ≥50 years, the prevalence was 56% in patients with MFS (19/34) vs 41% in controls (14/34) (*P* < 0.05) (Figure 2, Central Illustration).

**Central Illustration:**
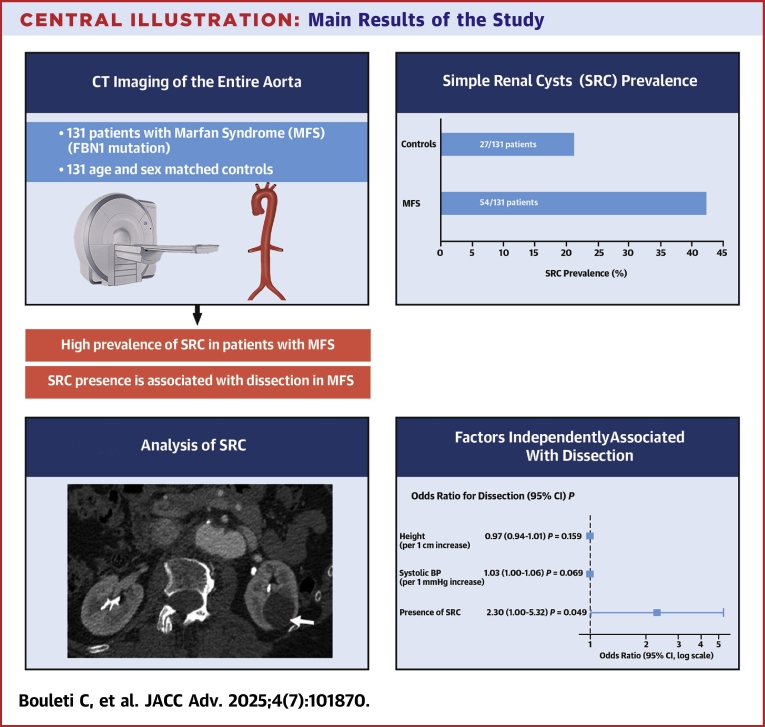
Main Results of the Study In 131 patients with MFS and their matched controls who underwent a CT-CAP at our institution, SRC prevalence was twice as high in the MFS group. The presence of SRC was independently and significantly associated with dissection in patients with MFS (adjusted OR: 2.30 [95% CI: 1.00-5.32]; *P* = 0.049). Abbreviations as in Figure 1, Figure 2.

There was no significant association between SRC and medication use (beta-blockers or angiotensin-converting enzyme inhibitor/angiotensin II receptor blockers).

### SRC prevalence and aortic disease severity

**Figure 3:**
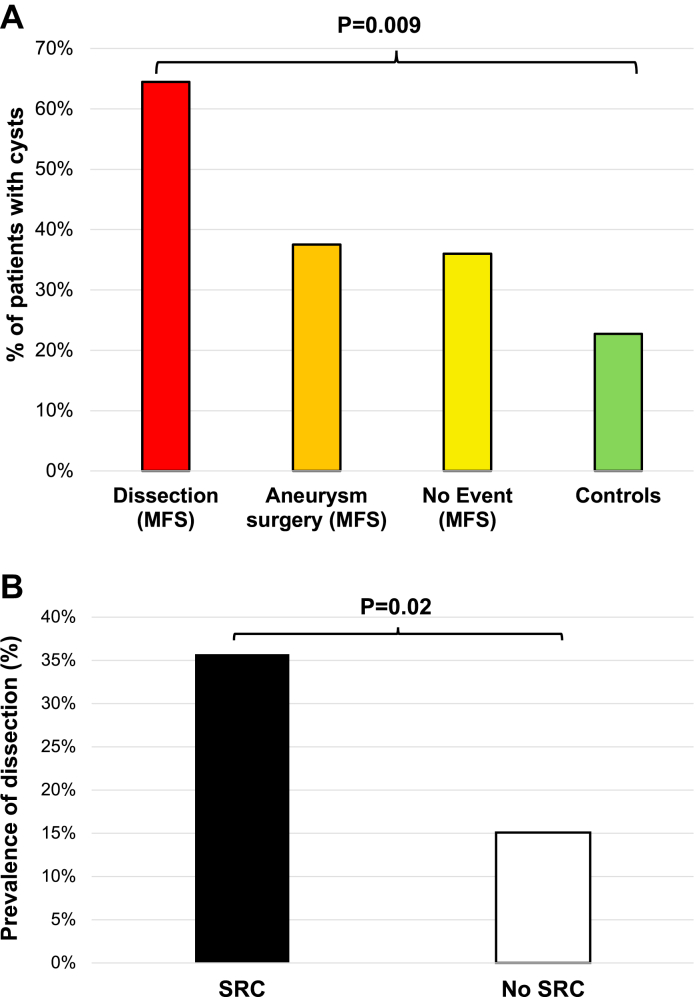
SRC and Aortic Events (A) Proportion of patients with SRC according to the severity of aortic disease. (B) Prevalence of arterial dissection in MFS patients with or without SRC. Abbreviations as in Figure 1, Figure 2.

We analyzed the proportion of patients with at least 1 SRC in the whole population of 262 patients, both patients with MFS and controls, according to the severity of aortic disease in patients with MFS only (as controls did not have aortic disease or events). As illustrated in Figure 3A, the prevalence of SRC was associated with the degree of aortic diseases/events. The most severe aortic event, dissection, was associated with the highest SRC prevalence (59%), followed by aortic surgery for aneurysm (43%). In contrast, the 32 MFS patients without an aortic event and the 131 controls had the lowest SRC prevalence (19% and 21%, respectively). The difference between groups was statistically significant (*P* = 0.009) (Figure 3A).

These findings were not associated with significant age differences between groups.

### Relationship between SRC and aortic complications in the population with MFS

Among the patients with MFS, we analyzed 54 patients with and 77 patients without SRCs, as detailed in Table 3.

**Table 3:** Characteristics of MFS Patients According to the Presence or the Absence of SRC

	Total MFS Population (n = 131)	With SRC (n = 54)	Without SRC (n = 77)	*P* Value
Age (y)	39.8 ± 13.8	43.8 ± 14.2	37.0 ± 12.9	0.005
Female	55 (42)	23 (43)	32 (42)	0.91
Height (cm)	184.7 ± 11.7	184.1 ± 12.4	185.1 ± 11.2	0.64
Weight (kg)	80.3 ± 17.9	80.4 ± 18.5	80.2 ± 17.6	0.96
Body mass index	23.6 ± 4.9	23.8 ± 5.2	23.4 ± 4.6	0.70
Body surface area (m^2^)	2.03 ± 0.24	2.03 ± 0.3	2.03 ± 0.2	1.00
Systolic BP (mm Hg)	127 ± 15	128 ± 15	127 ± 15	0.85
Diastolic BP (mm Hg)	73 ± 10	73 ± 10	73 ± 10	0.75
Heart rate (beats/min)	61 ± 12	60 ± 12	62 ± 12	0.25
Systemic Ghent-2 score (points)	6.69 ± 3.82	6.39 ± 3.57	6.91 ± 3.99	0.44
Aortic surgery for aneurysm	67 (51)	29 (54)	38 (49)	0.78
Aortic dissection	32 (24)	19 (35)	13 (17)	0.02
Right kidney width (mm)	52.2 ± 7.5	52.4 ± 8.4	52.1 ± 7.0	0.80
Right kidney length (mm)	108.9 ± 11.8	110.1 ± 12.3	108.2 ± 11.4	0.36
Left kidney width (mm)	54.5 ± 7.2	53.6 ± 8.1	55.1 ± 6.4	0.24
Left kidney length (mm)	114.1 ± 18.4	113.4 ± 22.3	114.6 ± 15.6	0.72

Values are mean ± SD or n (%).

Abbreviations as in Table 1, Table 2.

We found 2 factors to be significantly different between groups: age; and the prevalence of aortic dissection, which was higher in patients with SRCs compared to those without.

There was no significant difference in overall phenotypic severity, as assessed by the systemic Ghent-2 score, between the groups.

The prevalence of surgery for thoracic aortic aneurysm was not significantly different between patients with SRCs and those without (54% vs 49%, respectively; *P* = 0.78).

However, the prevalence of dissection was twice as high in patients with SRCs compared to those without (35% vs 17%; *P* = 0.02) (Figure 3B). The number of SRCs was not associated with the prevalence of dissection (*P* = 0.17). The types of dissection were as follows: type A aortic dissection in 18 patients, type B in 14. In univariate analysis, age, height, systolic blood pressure, and SRC were associated with aortic dissection (adjusted OR: 2.41 [95% CI: 1.08-5.45]; *P* = 0.033 for the presence of SRC) (Central Illustration, Supplemental Table 1). Age had a higher variance inflation factor value than SRC, and a significant interaction was observed between age and SRC (r = 0.28; *P* = 0.001) (Supplemental Tables 1 to 3). We therefore conducted 2 multivariable models: one including SRC and age, and the other including SRC only. In the first model, none of the parameters were significantly associated with dissection, while in the second model, only the presence of SRC was significantly and independently associated with aortic dissection (adjusted OR: 2.30 [95% CI: 1.00-5.32]; *P* = 0.049) (Supplemental Figure 2, Supplemental Table 4).

## Discussion

### Increased SRC prevalence in aortic diseases

Several studies have reported an increased prevalence of SRC in case of thoracic aortic aneurysms or dissection in the general population.^2^, ^3^, ^4^ Kim et al reported a significant increase in SRC prevalence, as assessed by CT scans, in 518 patients with aortic dissection compared to 1,366 healthy controls (*P* < 0.05).^2^ The most recent retrospective study, conducted on 35,498 patients with CT scans, reported that SRCs were significantly associated with the presence of aortic disease (OR: 2.57).^4^ Similarly, Ziganshin et al retrospectively analyzed 842 patients with an aortic disease (aneurysm or dissection) between 2004 and 2013 and compared them to 543 control patients. They observed a 3-fold increase in SRC prevalence in the aortic disease group compared to controls, *P* < 0.0001.^3^ In this series, only 18 patients (2%) had MFS, and no increased SRC prevalence was reported in this subset; however, the limited sample size precluded any definitive conclusion. Moreover, autosomal polycystic kidney disease has also been associated with a higher prevalence of aortic aneurysms.^22^ Finally, a recent preclinical study in a murine Marfan model revealed an association between renal cystic disease and enhanced aortic aneurysm formation.^7^

To date, only one study has analyzed SRC prevalence in 69 clinically diagnosed MFS patients and reported a higher prevalence compared to controls.^23^ This difference was statistically significant only in patients aged 40 to 60 years, likely due to a limited statistical power from small sample size. The major limitation of this study was the absence of genetic confirmation in patients, as the clinical diagnosis of MFS alone does not ensure a homogeneous population.^24^^,^^25^

Moreover, a number of pathogenic variants in genes other than *FBN1*, including *TGFBR1, TGFBR2, TGFB2, SMAD3,* and *ACTA2,* have been reported in other genetic conditions associated with aortic disease, potentially leading to different clinical presentations.^26^

The present study reports for the first time an increased prevalence of SRC in a homogeneous MFS population with identified *FBN1* pathogenic variants. Furthermore, the presence of SRC was associated with the severity of the aortic impairment, particularly with a significantly higher prevalence of dissection. While sex differences have been documented in MFS, we found no correlation between sex and SRC prevalence or the severity of aortic disease in our cohort.^27^, ^28^, ^29^

These findings, observed in the general population with aortic diseases, in patients with polycystic kidney disease, and in patients with MFS, raise the question of a shared pathophysiology between renal and aortic wall fragility.

### SRC and structural aortic diseases: a shared pathophysiology?

The pathophysiological link between aortic disease and SRC is not yet elucidated but different hypotheses have been raised, including structural weakness of both arterial and renal cyst walls due to ECM impairment.^2^ MMPs may play a role in ECM defects by cleaving ECM proteins essential for wall integrity. Supporting this hypothesis, increased MMP activity has been reported in case of aortic aneurysm or dissection compared to control subjects.^5^^,^^10^ Elevated MMP levels have also been measured in renal cyst fluid.^6^ Furthermore, experimental treatment with MMP inhibitors significantly reduced the number of cysts in a murine model.^30^

MFS with *FBN1* pathogenic variants is a rare genetic disease involving genetically driven ECM disruption and arterial wall fragility due to impaired *FBN1* protein synthesis.^9^ This results in elevated levels of active TGF-β, leading to up-regulation of MMPs, and the development of aortic aneurysms and/or dissection.^31^, ^32^, ^33^, ^34^ Additionally, TGF-β1 has been reported to be overexpressed in cystic cells compared to normal kidney cells.^35^

Although current evidence is limited, SRC and aortic disease may share a common underlying wall vulnerability.

### Association between SRC and aortic events in patients with MFS

We report for the first time an association between the presence of SRC and the severity of aortic events in patients with MFS.

SRC prevalence was higher in patients with aortic aneurysm surgery compared to those free of aortic events, with the highest prevalence observed in patients with dissection, reaching 59% despite a young mean age of 41 years, as illustrated in Figure 3. Patients with SRCs were more likely to experience aortic dissection compared to those without SRCs, with a more than 2-fold higher risk (OR: 2.41; *P* = 0.033). In multivariable analysis, SRCs were significantly and independently associated with dissection, although with borderline significance, likely due to a lack of statistical power from limited sample size (adjusted OR: 2.30; *P* = 0.049).

In contrast, patients with MFS free of aortic events had a prevalence of SRC comparable to controls (approximately 20%).

SRC could thus serve as a marker of aortic wall the fragility. While these findings need confirmation in larger prospective studies, they may open new perspectives for risk stratification in patients with aortic diseases. Current guidelines recommend imaging (CT scan or magnetic resonance imaging) of the entire aorta in MFS,^20^^,^^21^^,^^36^ which includes the kidneys at the level of the abdominal aorta. Screening for SRCs would therefore require no additional examination.

### Study Limitations

This is a retrospective monocentric study on a sample size that may appear modest. However, MFS is a rare disease, and the studied population exclusively comprised patients with identified *FBN1* pathogenic variants, representing the largest study to date. Moreover, patients were included at the national reference center for MFS, covering patients from all over France. Although unlikely, we cannot exclude that some control patients may carry a pathogenic *FBN1* variant without having been clinically identified as MFS patients. As kidney function data were not collected, any potential association with the presence of SRC could not be assessed. While pregnancy is a known risk factor for aortic dissection in patients with MFS, pregnancy data were not available at the time of the study and therefore could not be accounted for in the multivariable analyses.

The prevalence of aortic events in this series was higher than those reported in previously published series.^29^ These results are influenced by a selection bias due to CT scan screening, as many patients with MFS underwent CT scans to monitor surgical outcomes or dissection. The main limitation is the absence of aortic diameter measurements, which would allow for an investigation of the relationship between SRC presence and aortic diameters. As previously acknowledged, most CT scans were obtained after aortic dissections that led to emergency surgery, and we chose not to rely on limited, partial data. Prospective studies with measurement of aortic diameter before the occurrence of aortic events are therefore necessary.

## Conclusions

This study demonstrates for the first time a higher prevalence of SRCs in MFS patients with identified *FBN1* pathogenic variants compared to matched controls. Notably, the presence of SRCs was associated with a higher frequency of dissection, and SRCs were independently associated with dissection in multivariable analysis. While these findings need to be validated in larger prospective studies, they raise the question of increased aortic wall vulnerability in the presence of SRCs and may support a closer follow-up of MFS patients with SRCs.

Perspective **COMPETENCY IN MEDICAL KNOWLEDGE:** In this study, the prevalence of SRCs was twice as high in 131 patients with MFS compared to their age- and sex-matched controls (42% vs 21%), with the difference remaining statistically significant across all age groups. Furthermore, the presence of SRCs was associated with the severity of aortic disease in MFS patients, and SRCs were the only independent factor associated with dissection. While SRCs have previously been linked to aortic diseases in the general population, this study represents the largest analysis conducted in MFS patients.

**TRANSLATIONAL OUTLOOK:** These findings indicate a potential shared pathophysiology between SRC and MFS, possibly involving dysregulation of the TGF-β pathway and increased MMP activity. Further prospective studies are warranted to determine whether SRC could represent a marker of aortic wall fragility in patients with MFS. If confirmed, SRC may be listed as a risk factor influencing clinical decision-making, including consideration for lowering surgical thresholds.

## Funding support and author disclosures

This study is supported by the 10.13039/501100005630French Society of Cardiology but there was no dedicated funding. The authors have reported that they have no relationships relevant to the contents of this paper to disclose.
