# Supplementary material for: Simple Renal Cysts in Marfan Syndrome: Prevalence and Association With Aortic Events
Source: JACC Adv. 2025 Jun 17;4(7):101870. doi: 10.1016/j.jacadv.2025.101870 (PMC12212261; doi:10.1016/j.jacadv.2025.101870)
Supplement: Supplemental_Material [file mmc1.pdf]

SUPPLEMENTARY MATERIAL

Supplementary Table 1 : Univariate analysis of the presence of aortic dissection in patients with MFS

Variables	Dissection (n=32)	No dissection (n=99)	Univariate Unadjusted OR and 95% CI	P	VIF
Age (years)	45.9 ± 14.0	37.8 ± 13.2	1.04 (1.01-1.07)	0.015	1,71
Male gender	18 (56)	58 (59)	0.98 (0.44-2.20)	0.953	3,29
Height (cm)	181.3 ± 13.0	185.7 ± 11.1	0.97 (0.94-1.00)	0.081	4,74
Weight (kg)	78.1 ± 16.9	80.9 ± 18.2	0.99 (0.97-1.01)	0.378	1,28
Body surface area (m^2)	2.0 ± 0.3	2.0 ± 0.2	0.30 (0.05-1.61)	0.170	3,27
Systolic BP (mmHg)	131 ± 15	126 ± 14	1.03 (1.00-1.06)	0.053	1,59
Diastolic BP (mmHg)	73 ± 9	73 ± 10	0.99 (0.94-1.03)	0.557	1,78
Heart rate (bpm)	64 ± 12	61 ± 12	1.02 (0.98-1.05)	0.359	1,32
Presence of SRC	17 (53)	30 (30)	2.41 (1.08-5.45)	0.033	1,17
Right kidney width (mm)	112.6 ± 24.2	114.8 ± 16.0	0.99 (0.97-1.02)	0.556	1,44
Right kidney length (mm)	54.0 ± 9.0	54.7 ± 6.5	0.99 (0.93-1.04)	0.605	1,95
Left kidney width (mm)	108.8 ± 11.8	109.1 ± 11.8	1.00 (0.97-1.03)	0.898	1,64
Left kidney length (mm)	52.0 ± 7.8	52.3 ± 7.4	0.99 (0.94-1.05)	0.845	1,86

Data are expressed as mean ± SD or numbers and percentages.

MFS : Marfan syndrome ; SRC : Simple renal cyst ; BP : Blood Pressure ; bpm : beat per minute; VIF: variance inflation factor

Supplementary Table 2: Spearman correlation r values between the variables tested in the relative risk model of aortic dissection in patients with MFS

	Male gender	Height (cm)	Weight (kg)	Body surface area (m^2)	Systolic BP (mmHg)	Diastolic BP (mmHg)	Heart rate (bpm)	Presence of SRC	Right kidney width (mm)	Right kidney length (mm)	Left kidney width (mm)	Left kidney length (mm)
Age (years)	-0,35	-0,45	0,14	-0,15	0,18	0,19	0,10	0,28	-0,05	-0,02	-0,17	-0,08
Male gender		0,77	-0,24	0,65	0,01	-0,12	-0,12	-0,07	0,41	0,35	0,36	0,42
Height (cm)			-0,24	0,74	-0,07	-0,08	-0,09	-0,11	0,39	0,28	0,31	0,37
Weight (kg)				-0,04	-0,01	0,03	0,13	-0,01	0,00	0,15	-0,07	0,11
Body surface area (m^2)					-0,01	0,09	-0,05	-0,06	0,39	0,46	0,32	0,47
Systolic BP (mmHg)						0,54	0,23	0,05	0,09	0,21	0,09	0,10
Diastolic BP (mmHg)							0,24	-0,06	0,06	0,09	0,04	0,05
Heart rate (bpm)								-0,13	-0,06	-0,13	0,01	-0,06
Presence of SRC									-0,05	-0,08	0,01	-0,03
Right kidney width (mm)										0,41	0,57	0,40
Right kidney length (mm)											0,35	0,51
Left kidney width (mm)												0,44

MFS: Marfan Syndrome; BP: Blood Pressure; SRC: Simple Renal Cyst

Supplementary Table 3: Spearman correlation p values between the variables tested in the relative risk model of aortic dissection in patients with MFS

	Male gender	Height (cm)	Weight (kg)	Body surface area (m^2)	Systolic BP (mmHg)	Diastolic BP (mmHg)	Heart rate (bpm)	Presence of SRC	Right kidney width (mm)	Right kidney length (mm)	Left kidney width (mm)	Left kidney length (mm)
Age (years)	0,0000	0,0000	0,1044	0,0953	0,0500	0,0364	0,2822	0,0011	0,5637	0,8077	0,0506	0,3585
Male gender		0,0000	0,0073	0,0000	0,8844	0,2024	0,1734	0,4066	0,0000	0,0000	0,0000	0,0000
Height (cm)			0,0070	0,0000	0,4645	0,3898	0,3213	0,1953	0,0000	0,0011	0,0003	0,0000
Weight (kg)				0,6356	0,8774	0,7671	0,1469	0,9221	0,9949	0,0814	0,4036	0,1953
Body surface area (m^2)					0,8874	0,3442	0,5760	0,5339	0,0000	0,0000	0,0002	0,0000
Systolic BP (mmHg)						0,0000	0,0094	0,5516	0,3451	0,0222	0,3119	0,2791
Diastolic BP (mmHg)							0,0073	0,5231	0,4812	0,3394	0,6777	0,5725
Heart rate (bpm)								0,1418	0,4873	0,1664	0,9289	0,4787
Presence of SRC									0,5936	0,3785	0,8944	0,7290
Right kidney width (mm)										0,0000	0,0000	0,0000
Right kidney length (mm)											0,0000	0,0000
Left kidney width (mm)												0,0000

MFS: Marfan Syndrome; BP: Blood Pressure; SRC: Simple Renal Cyst

Supplementary Table 4 : Multivariable analysis of the presence of aortic dissection in patients with MFS in both models

Variables	Adjusted OR and 95%CI (Model 1)	P	Adjusted OR and 95%CI (Model 2)	P
Age (per 1 year increase)	1.03 (1.00-1.06)	0.054		
Presence of SRC	1.91 (0.81-4.48)	0.136	2.30 (1.00-5.32)	0.049

MFS: Marfan Syndrome; SRC: Simple Renal Cyst

Supplementary Figure 1: **CT-scan images of renal cysts.**

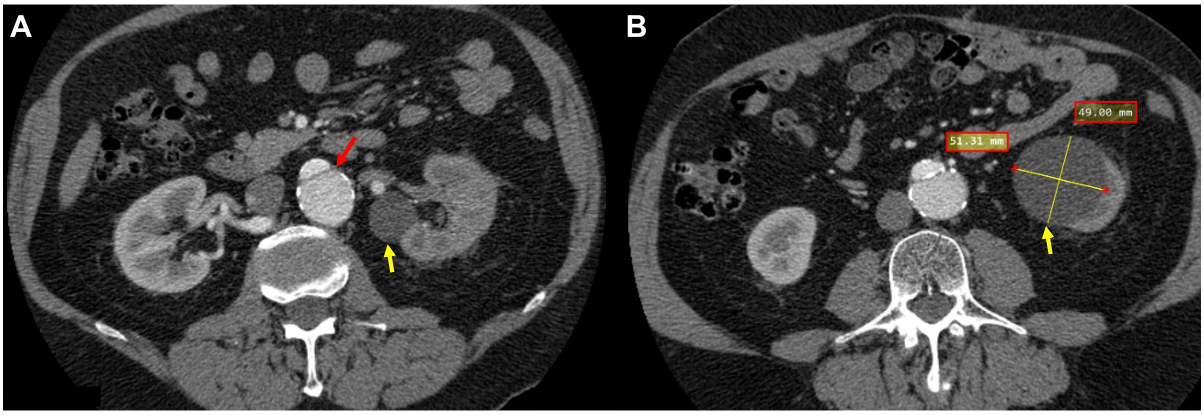

Polar superior and inferior (yellow arrows) (Panel A & B) left renal cysts in a Marfan patient with type B aortic dissection (red arrow)

Supplementary Figure 2: Forrest plot of the multivariable analysis for aortic dissection among patients with MFS

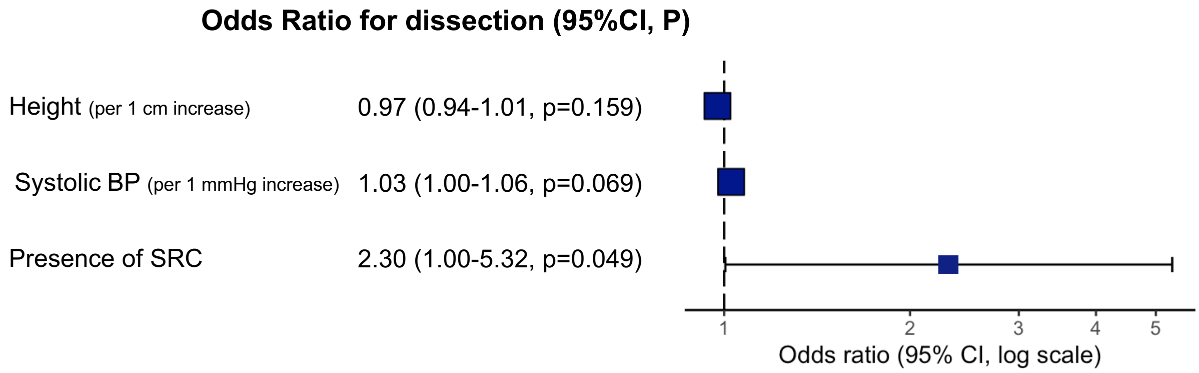

MFS: Marfan Syndrome; BP: Blood Pressure; SRC: Simple Renal Cyst
